# TRIM30 modulates Interleukin-22-regulated papillary thyroid Cancer cell migration and invasion by targeting Sox17 for K48-linked Polyubiquitination

**DOI:** 10.1186/s12964-019-0484-6

**Published:** 2019-12-10

**Authors:** Wei Li, Fen Li, Weiwei Lei, Zezhang Tao

**Affiliations:** 1 Department of Otolaryngology Head and Neck Surgery, Remin Hospital of Wuhan University, Wuhan, 430060 Hubei Province the, People’s Republic of China; 2 Research Institute of Otolaryngology-Head and Neck Surgery, Renmin Hospital of Wuhan University, Wuhan, 430060 Hubei Province the, People’s Republic of China

**Keywords:** Interleukin-22, Papillary thyroid cancer, Tripartite-motif protein 30, Sox17, β-Catenin, TRIM30, Follicular thyroid cancer

## Abstract

**Background:**

Interleukin-22 (IL-22) belongs to the IL-10 cytokine family and is mainly produced by activated Th1 cells. Although IL-22 expression is reported to be elevated in many cancers, and increased IL-22 expression correlates with tumor progression and poor prognosis, little is known about the role of IL-22 in papillary thyroid cancer (PTC). We previously demonstrated that IL-22 promotes PTC cell migration and invasion through the microRNA-595/Sox17 axis.

**Methods:**

We used qRT-PCR and western blot to determine TRIM30, Sox17 and β-catenin expression in PTC cells. Knockdown and overexpression were performed to detect the role of TRIM30/Sox17/β-catenin axis on the migration and invasion PTC cells. Co-IP were used to determine the interaction between TRIM30 and Sox17.

**Findings:**

In this study, we demonstrated that IL-22 triggered tripartite-motif protein 30 (TRIM30) association with Sox17, thereby mediating K48-linked polyubiquitination of Sox17. We then demonstrated that TRIM30 was a positive regulator of IL-22-regulated migration and invasion of PTC cells. We also found that IL-22 induced the transcriptional activity of β-catenin and translocation of β-catenin from cytosol to the nucleus. Upon investigating the mechanisms behind this event, we found that IL-22 disrupted Sox17/β-catenin interactions by inducing TRIM30/Sox17 interactions, leading to promotion of β-catenin-dependent signaling. The analysis of hundreds of clinical specimens revealed that IL-22, TRIM30 and β-catenin levels were upregulated in PTC tissues compared with normal thyroid, and that their expression levels were closely correlated. Taken together, under the influence of IL-22, by sequestration of Sox17, TRIM30 promotes β-catenin-dependent signaling that promotes PTC cell proliferation.

## Highlights

- IL-22 promotes PTC cells proliferation by TRIM30.
- TRIM30 interacts with Sox17.
- TRIM30 promotes β-catenin signaling by interacting and degrading Sox17.
- IL-22 promotes β-catenin signaling by inducing TRIM30/Sox17 interactions and disrupting Sox17/β-catenin interactions.

## In brief

Li et al. demonstrate that TRIM30-mediated IL-22 promoted PTC cells proliferation. Mechanistically, TRIM30 promotes PTC progression by interacting with Sox17 and by increasing β-catenin activity. High levels of TRIM30 were observed in patients, correlated with several malignant characteristics in PTC patients.

## Introduction

Thyroid carcinoma is the most frequent type of endocrine cancer, and the incidence of thyroid cancer is increasing worldwide [1, 2]. The majority of thyroid cancers are well-differentiated, with papillary thyroid carcinoma (PTC) and thyroid carcinoma (FTC) constituting the most common types [3, 4]. PTC is the most common subtype, in approximately 75–85% of patients [5]. FTC is the second most prevalent subtype, accounting for 10–15% of thyroid cancers [5]. Although most patients with well-differentiated cancer have favorable prognoses, overall recurrence rates may be as high as 35% [5]. The development of recurrence is associated with higher mortality [6]. Many studies found that tumor differentiation, aggressiveness and metastatic potential correlate with oncogenic alterations [7, 8]. Although some oncogenic genes have been extensively studied, other factors, particularly factors mediating the interactions between tumors and the surrounding microenvironment, have not been fully characterized. PTCs often display immune cells and desmoplastic stromal infiltrates associated with increased proinflammatory cytokine expression [9, 10]; nevertheless, the precise role of inflammatory mediators is not yet known.

Interleukin-22 (IL-22) is a member of the IL-10 family [11]. It is the signature cytokine of T-helper (Th) 22 cells [12]. IL-22 has recently been shown to be involved in the pathogenesis of autoimmune and inflammatory disorders, including psoriasis, lupus erythematosus, and rheumatoid arthritis [13, 14]. IL-22 binds to cell surface heterodimeric receptor complexes composed of IL-22RA1 and IL-10R2, thereby activating STAT3 and MAPK [15]. IL-10R2 is ubiquitously expressed; however, IL-22RA1 expression is limited to epithelial cells in the digestive system, skin, and respiratory tree [16]. IL-22 receptor distribution leads to IL-22 having a direct impact on epithelial cells; however, with little or no interaction with immune cells [17]. Because of the role of IL-22 in epithelial cells, IL-22 thought to be beneficial for intestinal epithelial barrier function because of increased epithelial cell proliferation, migration, and mucus production [18]. Importantly, many studies found that IL-22 was closed related to progression and metastasis of many cancer, including breast, lung, and gastric cancer [19, 20]. Nevertheless, a clear role for IL-22 in progression and metastasis of PTC has not been established, and the molecular mechanisms by which IL-22 regulates progression of PTC are ill-defined.

In our previous study, we found that IL-22 promoted the migration and invasion of PTC cells, and sex-determining region Y-box 17 (Sox17) was involved [21]. In this study, we demonstrated that tripartite-motif protein 30 (TRIM30) interacts with Sox17 and thereby impairs the Sox17/β-catenin interaction, leading to promotion of translocation of β-catenin from cytosol to the nucleus. We further demonstrated that IL-22 regulated β-catenin signaling and migration and invasion of PTC cells via the TRIM30/Sox17 axis.

## Materials and methods

### Ethics statement

The study was conducted according to the principles of the Declaration of Helsinki and approved by the Institutional Review Board of the Remin Hospital of Wuhan University, in accordance with its guidelines for the protection of human subjects. All study participants provided written informed consent for the collection of samples and subsequent analyses.

### Samples and cases

Thyroid tissues were collected at the Hubei Cancer Hospital and the Renmin Hospital of Wuhan University, from April 2010 to April 2019. Tissue samples were cut into two parts; one was reviewed by two expert pathologists to verify the histologic diagnosis, the other immediately snap-frozen in liquid nitrogen, and stored in liquid nitrogen until RNA extraction. None of the patients had received any preoperative treatment. Tumors were staged according to the American Joint Committee on Cancer (AJCC) pathologic tumor-nodemetastasis (TNM) classification. The characteristics of patients are described in Additional file 1: Table S1.

### Cell, reagents and constructs

Human papillary thyroid cancer cell lines K1 and TPC-1 were obtained from CCTCC (China Center for Type Culture Collection). All cells were grown in Dulbecco’s modified Eagle’s medium supplemented with 10% heat-inactivated fetal bovine serum, 100 U/mL penicillin, and 100 μg/mL streptomycin sulfate at 37 °C in 5% CO_2_.

TRIzol, Lipofectamine-3000, and Enzyme MIX were purchased from Invitrogen (Basel, Switzerland). The details of antibodies are listed in Additional file 1: Table S2. ShRNAs and negative control (shRNA-control) were purchased from Qiagen (Germany) (Additional file 1: Table S3). Efficiency of RNA interferences was determined using qRT-PCR and western blotting analyses (Additional file 1: Fig. S1A). Recombinant human IL-22 (rhIL-22) was purchased from R&D Systems (Minneapolis, MN). Chemically synthesized micro RNA (miRNA) mimics and miRNA inhibitors were purchased from Ambion (Catalog # 4464066) (Austin, TX, USA). Unless specified otherwise, all biochemical reagents were purchased from Sigma-Aldrich.

Sequences encoding TRIM30α, β-catenin and Sox17 were amplified by PCR with cDNA from BMDCs challenged for 4 h with LPS. TRIM30αand deletion mutants were cloned into pCMV-Flag2B vector (Invitrogen). Sox17 and deletion mutants were cloned into pcDNA3.1(−)-Myc-His vector (Invitrogen). β-catenin were cloned into pcaggs-HA vector (Invitrogen). HA-Ub (WT), HA-Ub (K63) and HA-Ub (K48) were gifts from Dr. Hongbing Shu (Wuhan University). β-catenin-luc was a gift from Randall Moon (Addgene plasmid # 12456). To verify constructs and the specificity of antibodies, all constructs were transfected into 293 T cells, and expression was analyzed using western blot. All constructs were confirmed by DNA sequencing (Sangon Biotech, Shanghai, China).

### Coimmunoprecipitation

Coimmunoprecipitation analyses were performed, as previously described [22]. Briefly, after treatment, cells were collected and lysed using lysis buffer (20 mM Tris, pH 7, 0.5% (vol/vol) Nonidet-P40, 25 mM NaCl, 3 mM EDTA, 3 mM EGTA, 2 mM dithiothreitol, 0.5 mM phenylmethyl sulfonyl fluoride, 20 mM β-glycerol phosphate, 1 mM sodium vanadate and 1 mg/ml of leupeptin). Lysates were mixed and were precipitated with antibodys or IgG and protein G-agarose beads by overnight incubation at 4 °C. Beads were washed three-five times with lysis buffer, and bound proteins were separated by SDS-PAGE with subsequent immunoblotting analysis.

### Immunopurification and mass spectrometry

TPC-1 cells were transfected with the vector control or Flag-Sox17 for 48 h. Anti-Flag immunoaffinity columns were prepared using anti-FLAG M2 affinity gel (Sigma) following the manufacturer’s suggestions. Cell lysates were obtained from about 5 × 10^8^ cells and applied to an equilibrated FLAG column of 1-ml bed volume to allow for adsorption of the protein complex to the column resin. After binding, the column was washed with cold PBS plus 0.1% NP-40. Flag peptide (Sigma) was applied to the column to elute the Flag protein complex as described by the vendor. Fractions of the bed volume were collected and resolved on SDS-polyacrylamide gel, silver stained, and subjected to LC-MS/MS sequencing and data analysis.

All MS/MS samples were analyzed using X! Tandem (the GPM, thegpm.org; version Alanine 2017.2.1.4). Scaffold (version Scaffold_4.8.4; Proteome Software) was used to validate MS/MS based peptide and protein identifications. Peptide identifications were accepted if they exceeded specific database search engine thresholds. X! Tandem identifications required at least -log (expect scores) scores of > 2.0 with a mass accuracy of 5 ppm. Protein identifications were accepted if they contained at least two identified peptides. Our threshold for peptide acceptance was > 95% probability.

### Nuclear extraction

Cells were incubated in serum-free media for 24 h, washed twice with cold PBS, and scraped into 1 ml cold PBS. Cells were harvested by centrifugation (15 s) and incubated in two packed cell volumes of buffer A (10 mM HEPES, pH 8, 0.5% Nonidet P-40, 1.5 mM MgCl_2_, 10 mM KCl, 0.5 mM DTT, and 200 mM sucrose) for 5 min at 4 °C with flipping of the tube. The crude nuclei were collected by centrifugation (30 s); pellets were rinsed with buffer A, resuspended in one packed cell volume of buffer B (20 mM HEPES, pH 7.9, 1.5 mM MgCl_2_, 420 mM NaCl, 0.2 mM EDTA, and 1.0 mM DTT), and incubated on a shaking platform for 30 min at 4 °C. Nuclei were centrifuged (5 min), and supernatants were diluted 1:1 with buffer C (20 mM HEPES, pH 7.9, 100 mM KCl, 0.2 mM EDTA, 20% glycerol, and 1 mM DTT). Cocktail protease inhibitor tablets were added to each type of buffer. Nuclear extracts were snap-frozen in liquid nitrogen and stored at − 70 °C until use.

### Cell proliferation, migration and invasion assays

The Cell Counting Kit-8 (CCK-8, Dojindo Chemical Laboratory, Kumamoto, Japan) and colony formation assay were conducted to determine proliferation activity. Approximately 5 × 10^3^ cells per well were transferred to 96-well plates after transfection and were subjected to the CCK-8 assay. After incubation at 37 °C for 1.5 h, the absorbance at 450 nm was measured following the addition of 10 μL CCK-8 solution. There were 5 replicates in each group, and 3 independent experiments were performed.

Migration and invasion assays were performed with a modified Boyden chamber (Corning) containing Matrigel-coated (invasion) or no Matrigel-coated (migration) membrane matrices (BD Biosciences). Cells were plated in the upper chamber, and the lower chamber contained medium with 10% FBS, and cells were incubated for 24 h. After fixation with 4% formaldehyde, cells on the lower surface of the membranes were stained with crystal violet and were observed under a microscope at 200x magnification. The average number of cells was determined from six representative fields.

### Luciferase reporter gene assays

Cells were seeded on 24-well dishes and transfected using Lipofectamine 3000 (Invitrogen) following the manufacturer’s recommended protocol. For β-catenin reporter assays, cells were transfected with 300 ng of β-catenin reporter plasmid, 30 ng of pTK-RL reporter vectors (Stratagene) and various amounts of the relevant expression plasmids as described in the figure legends, maintaining the total amount of DNA constant using empty vector. Twenty-four hours after transfection, cells were serum-starved for an additional 24 h prior to harvest. Luciferase assays were performed using a dual-specific luciferase assay kit (Promega, Madison, WI). Firefly luciferase activities were normalized on the basis of Renilla luciferase activities.

### Generation of knockout cell lines

The lentiCRISPRv2 plasmid was a gift of Professor Shi Liu (Wuhan University). A specific oligo targeting the gene was designed using the Cas9 target design tools (http://www.genome-engineering.org). The designed oligos are listed in Additional file 1: Table S3. The target guide sequence cloning protocol can be found at the Zhang Laboratory GeCKO Web site (http://www.genome-engineering.org/gecko/). The specific lentiCRISPRv2 plasmid, lentivirus packaging plasmid psPAX2, and envelope plasmid pMD2.G were cotransfected into 293 T cells in 60-mm culture dishes using Lipofectamine 3000. The harvested medium was centrifuged at 15,000×g for 5 min and then filtered through a 0.22-mm filter (Millipore) to remove cells. When cells were grown to ∼70% confluence, they were incubated in fresh culture medium containing 8 mg/ml polybrene. Subsequently, we added specific lentiCRISPRv2 lentivirus-containing media to the cells. The monoclonal cells were singled out for enlarged culture. KO cell lines were obtained from these enlarged monoclonal cells, and KO cells were confirmed by qRT-PCR and western blotting (Additional file 1: Fig. S1B).

### Quantitative real-time RT-PCR (qRT-PCR)

Total RNA was extracted using TRIzol reagent (Invitrogen). cDNA was synthesized with the Prime-Script RT reagent Kit (TaKaRa). The expression of mature miRNAs was assayed using TaqMan MicroRNA Assays (Applied Biosystems, Foster City, CA, USA). A two-step qRT-PCR was employed with specific primers for miRNAs designed by Applied Biosystems. U6 small nuclear RNA (snRNA) was amplified as an internal control. Real-time PCR analyses for TRIM30, β-catenin, Sox17 and GAPDH were performed using SYBR Premix Ex Taq (Takara Bio, Dalian, China). The primers used were listed in Additional file 1: Table S3. Real-time PCR was performed using the ABI 7900 real-time PCR machine. The relative expression of each gene was calculated and normalized using the 2^−ΔΔCt^ method.

### Xenograft tumor model

All animal experiments were performed in accordance with the National Institutes of Health Guide for the Care and Use of Laboratory Animals. The protocol was approved by the institutional animal care and use committee of Remin Hospital of Wuhan University. For xenograft experiments, ten 6-week-old male BALB/c nude mice were divided into groups randomly. Each group was composed of 6 mice that were injected with 2 × 10^6^ indicated cells. Tumor volume was calculated by measuring the length and width of the tumor (tumor volume = 1/2 length×width^2^) every week. After 5 weeks, the mice were sacrificed by anesthesia and the tumors were removed and weighed for further analysis.

### Western blot analysis

Western blot analyses were described in a previous study [23]. Briefly, cells were harvested by low-speed centrifugation and washed with PBS. Cells were lysed in RIPA Buffer (Cell Signaling Technology, 9800), and protein concentrations were determined using BCA assays (Cell Signaling Technology, 7780). Forty micrograms of each protein sample were separated using 12% SDS-PAGE and transferred to nitrocellulose membranes (Bio-Rad). Membranes were blocked with 1 × Tris-buffered saline with Tween 20 (TBST) and 5% (w/v) non-fat milk for 1 h at room temperature. Then, membranes were incubated with primary antibodies overnight at 4 °C. Subsequently, blots were incubated with horseradish peroxidase-linked secondary antibodies (Jackson ImmunoResearch) for an additional 1 h. Immunoreactive bands were visualized using an enhanced chemiluminescence system (GE Healthcare).

### Statistical analysis

Statistical analyses were performed using the GraphPad Prism 5 software (GraphPad Software, La Jolla, CA, USA). Parametric and nonparametric data were analyzed using a two-tailed t test and the Mann-Whitney U test respectively. A value of *P* < 0.05 was considered statistically significant. Data are presented as mean ± SD or mean ± SEM.

## Results

### Identification of TRIM30 as a Sox17-associated protein

**Fig. 1:**
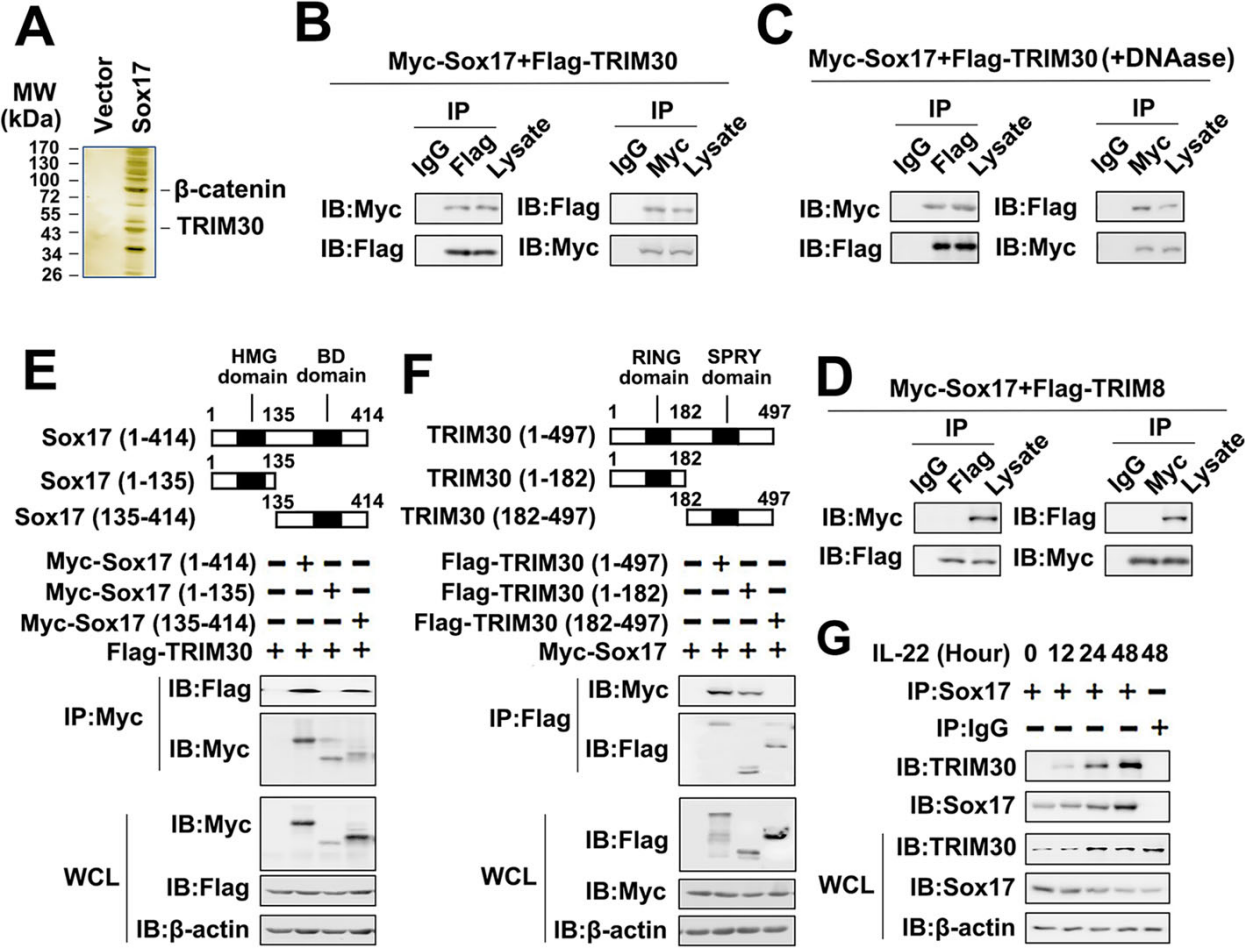
TRIM30 associates with Sox17. (**a**) TPC-1 cells were transfected with vector control or Myc-Sox17 for 48 h. Cells were lysed and immunopurified with anti-Flag affinity columns and eluted with Flag peptide. The eluates were resolved using SDS-PAGE and were silver-stained. The various protein bands were retrieved and analyzed using mass spectrometry. (**b**) 293 T cells were transfected with Flag-tagged TRIM30 (Flag-TRIM30) and Myc-tagged Sox17 (Myc- Sox17). Forty-eight hours post-transfection, Co-IP and immunoblot analysis were performed with the indicated antibodies. (**c**) Experiments were performed in similar fashion to those in (**b**), except DNAase was used. (**d**) Experiments were performed in similar fashion to those in (**b**), except Flag-TRIM8 was used. (**e**) Schematic diagram of the full-length and truncated constructs of Sox17 (upper panel). 293 T cells were co-transfected with Flag-TRIM30 and the indicated truncated Sox17 constructs for 48 h. Co-IP and immunoblot analyses were performed with the indicated antibodies (lower panel). (**f**) Experiments were performed in similar fashion to those in (**e**), except indicated truncated constructs of TRIM30 were used. (**g**) TPC-1 cells were treated with rhIL-22 at 50 ng/ml for indicated times. Immunoprecipitation and immunoblot analysis were performed with the indicated antibodies. All experiments were repeated at least three times

In a previous study, we found that Sox17 played an important role in IL-22-regulated PTC cell migration and invasion [21]. To further explore the molecular mechanisms by which Sox17 exerts its tumor-inductive effects on PTC cells, we used immunoprecipitation coupled to mass spectrometry (IP-MS) to identify binding partners for Sox17 (Additional file 1: Table S4). We found that TRIM30 was a potential target of Sox17 (Fig. 1a). Co-immunoprecipitation (Co-IP) and reverse Co-IP experiments were performed to further confirm the binding of the TRIM30 to Sox17. As shown in Fig. 1b, Myc-tagged Sox17 (Myc-Sox17) interacted with Flag-tagged TRIM30 (Flag-TRIM30). Interesting, DNAase failed to affect Sox17/TRIM30 interaction, suggesting that Sox17 and TRIM30 interaction not mediated by DNA (Fig. 1c). By contrast, another TRIM-containing protein in the TRIM family, TRIM8, did not interact with Sox17 (Fig. 1d). To map the region of Sox17 that interacted with TRIM30, we constructed a series of Sox17 plasmids with Myc-tagged truncation mutants (Fig. 1e, upper panel). We demonstrated that the C-terminal domain of Sox17 was necessary for its interaction with TRIM30 (Fig. 1E, lower panel). Similarly, the N-terminal domain of TRIM30 were required for its binding to Sox17 (Fig. 1f). We further performed endogenous Co-IP experiments, and found that Sox17 weakly associated with TRIM30 in untreated cells. This association increased after treatment with IL-22 (Fig. 1g). Taken together, these data suggest that Sox17 is associated with TRIM30.

### IL-22 regulatd Sox17 ubiquitination through TRIM30

**Fig. 2:**
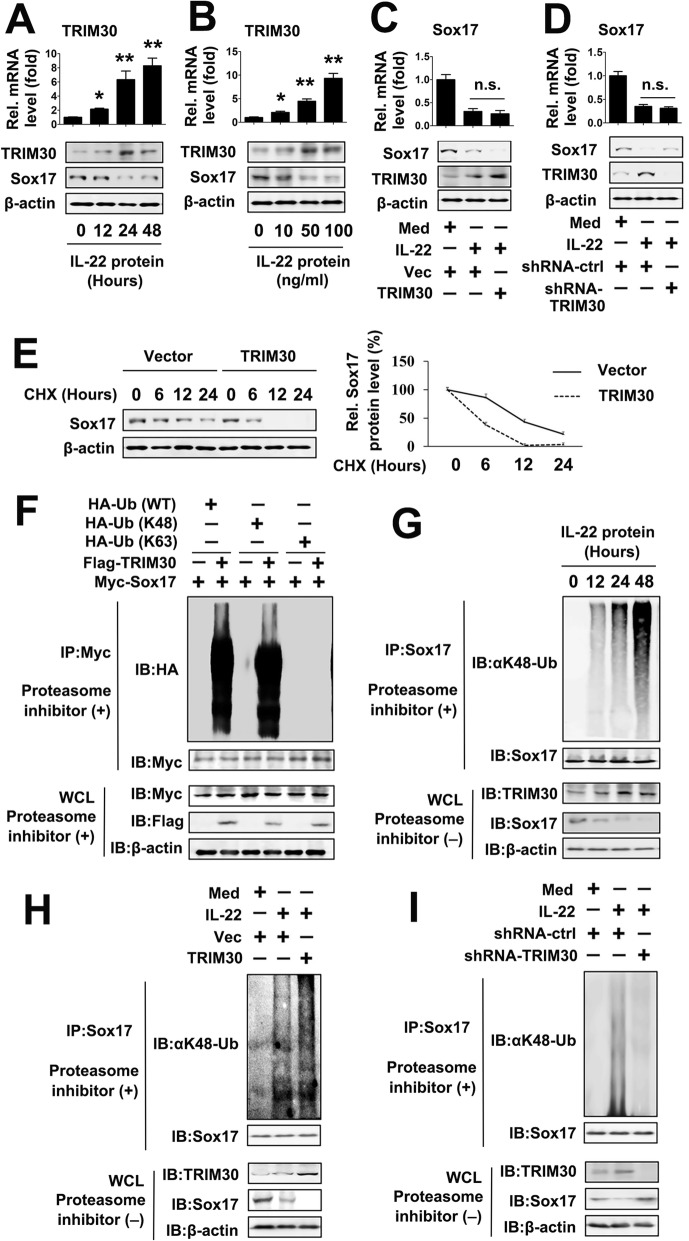
TRIM30 is involved in IL-22 regulated K48-linked polyubiquitination of Sox17. (**a**) TPC-1 cells were treated with 50 ng/ml rhIL-22 for the indicated time points. TRIM30 RNA levels were quantified using qRT-PCR (upper panel) and protein levels of TRIM30 and Sox17 were measured using western blot (lower panel). (**b**) TPC-1 cells were treated with rhIL-22 for 24 h at indicated concentrations. TRIM30 RNA levels were quantified using qRT-PCR (upper panel) and protein levels of TRIM30 and Sox17 were measured using western blot (lower panel). (**c**) TPC-1 cells were transfected with indicated plasmids for 24 h and treated with or without 50 ng/ml rhIL- 22 for 24 h prior to real-time RT-PCR (upper panel) and western blot (lower panel). (**d**) Experiments were performed as in (**c**) except cells were transfected with TRIM30-specific shRNA (shRNA-TRIM30). (**e**) TPC-1 cells were transfected with pCMV-TRIM30 or control vector for 48 h and treated with or without cycloheximide (CHX) for indicated times prior to western blot. (**f**) Overexpressed TRIM30 enhances wild-type and K48-linked polyubiquitination of Sox17. A total of 293 cells (2 × 10^6^) were transfected with Sox17 or/and Flag-TRIM30 and the indicated ubiquitin plasmids for 24 h and treated with MG132 (100 μM) for 6 h. Cell lysates were immunoprecipitated with anti-Myc. The immunoprecipitates were analyzed by immunoblots with anti-HA, anti-Myc, or anti-Flag as indicated. (**g**) TPC-1 cells were treated with 50 ng/ml rhIL-22 for the indicated time points. Co-IP and immunoblot analyses were performed with the indicated antibodies. (**h**) TPC-1 cells were transfected with indicated plasmid for 24 h and treated with or without 50 ng/ml rhIL- 22 for 24 h. Co-IP and immunoblot analyses were performed with the indicated antibodies. (**i**) Experiments were performed as in (**c**) except cells were transfected with shRNA-TRIM30. All experiments were repeated at least three times. Bar graphs present means ± SD, *n* = 3 (**P < 0.01; *P < 0.05)

Because TRIM30 interacts with Sox17 under IL-22 treatment, we investigated whether IL-22 affected the expression of TRIM30. A time-dependent increase TRIM30 expression was observed in TPC-1 cells treated with increasing time-exposure of IL-22 (Fig. 2a). Similarly, increased TRIM30 expression was also detected in TPC-1 cells treated with increasing concentrations (from 10 to 100 ng/ml) of IL-22 over 24 h (Fig. 2b). We next investigated whether TRIM30 affected IL-22-regulated Sox17 expression. We found that TRIM30 overexpression inhibited IL-22-regulated Sox17 protein levels, but did not affect mRNA levels (Fig. 2c). We designed two shRNAs for TRIM30 (TRIM30 shRNA #1 and #2) and tested their efficiency (Additional file 1: Fig. S1A). ShRNA-TRIM30#2 was selected for experiments described below. TRIM30 knockdown also increased IL-22-regulated Sox17 protein levels, but not mRNA levels (Fig. 2d). Consistent with these observations, the half-life of Sox17 was lower in TRIM30-overexpressing TPC-1 cells than in TPC-1 cells expressing empty vector (Fig. 2e). In an overexpression system, TRIM30 enhanced K48-linked, but not K63-linked, polyubiquitination of Sox17 (Fig. 2F). Furthermore, endogenous Co-IP experiments showed that time-dependent K48-linked ubiquitination of Sox17 was greater in IL-22 treated cells than in control cells (Fig. 2G). Interestingly, TRIM30 overexpression increased IL-22-induced Sox17 ubiquitination, whereas TRIM30 knockdown decreased IL-22-induced Sox17 ubiquitination (Fig. 2h and i). These data suggest that TRIM30 interacts with and ubiquitinates Sox17, and IL-22-induces Sox17 ubiquitination via TRIM30.

### IL-22 induces papillary thyroid cancer cell proliferation via the TRIM30/Sox17 axis

**Fig. 3:**
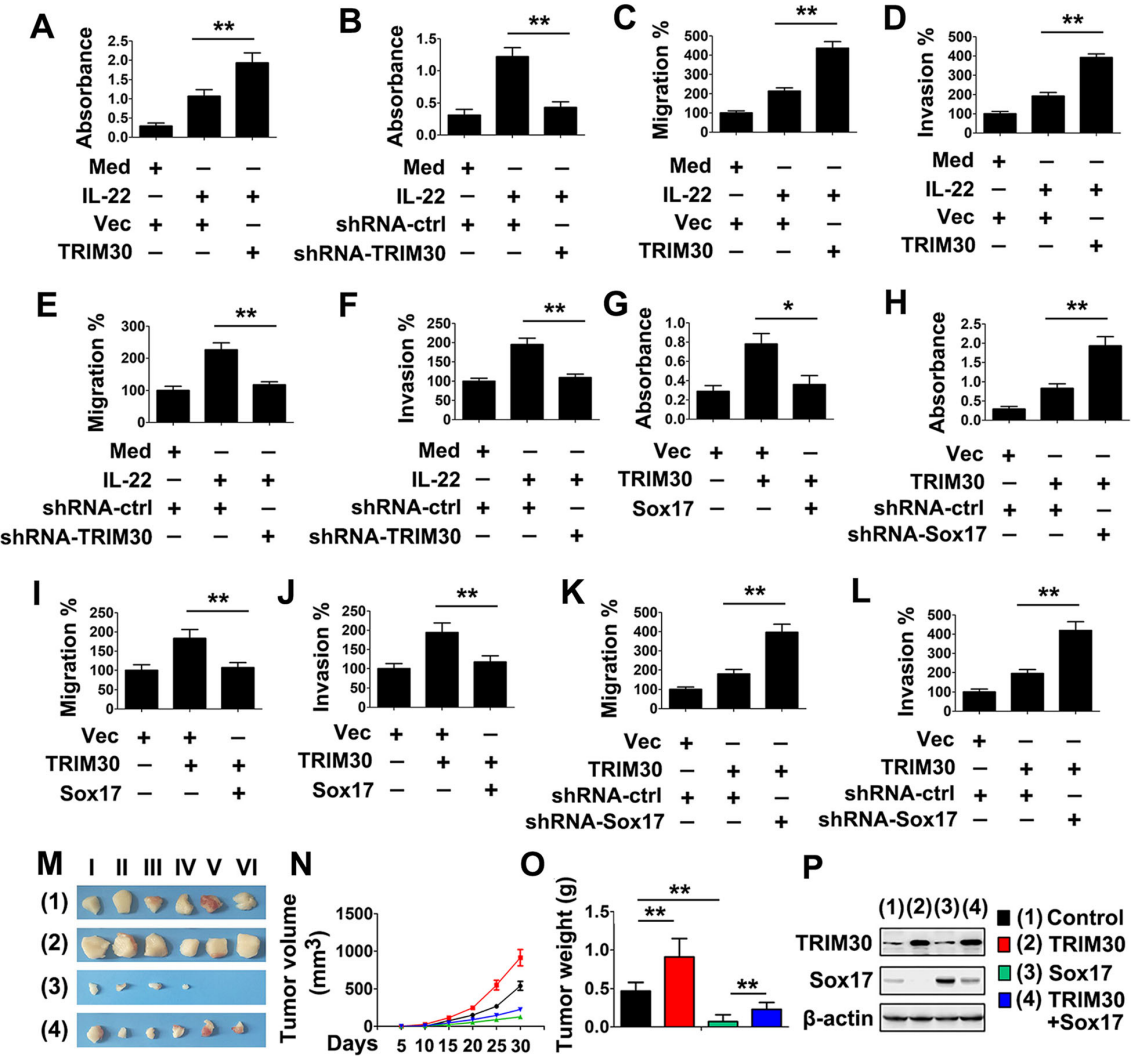
IL-22 promotes cell growth and motility via TRIM30/Sox17 axis. (A) TPC-1 cells transfected with the pCMV-TRIM30 or control vector for 24 h and treated with or without 50 ng/ml rhIL- 22 for 24 h prior to cell proliferation assay. (B) Experiments were performed as in (**a**), except cells were transfected with shRNA- TRIM30. (C and D) TPC-1 cells were transfected with the indicated plasmid, then treated with rhIL-22 (50 ng/ml) for 48 h prior to Transwell migration (**c**) or invasion (**d**) assays. (**e** and **f**) Experiments were performed as in (**c** and **d**), except cells were transfected with shRNA-TRIM30. (G) TPC-1 cells were transfected with the plasmids as indicated for 48 h prior to cell proliferation assay. (**i**) Experiments were performed as in (**g**), except cells were transfected with shRNA-Sox17. (I and J) TPC-1 cells were transfected with the plasmids as indicated for 48 h prior to Transwell migration (**i**) or invasion (**j**) assays. (**k** and **l**) Experiments were performed as in (I and J), except cells were transfected with shRNA-Sox17. (M-P) TPC-1 cells were stably expressing TRIM30, SOX17, or TRIM30 and SOX17 were subcutaneous injected into nude mice. Shown are stripped tumors (**m**), the growth curve (**n**), and the average weight (**o**). The indicated tumor tissue from a single mouse in each group was cut into several pieces and then used for immunoblot (**p**). Tumors were from the fourth column in each group. Bar graphs present means ± SD, *n* = 3 (**P < 0.01; *P < 0.05)

Because Sox17 suppresses IL-22 induced migration and invasion of PTC cells and TRIM30 interacts with Sox17, we reasoned that TRIM30 was also involved in the migration and invasion of PTC cells. As shown in Fig. 3a, TRIM30 overexpression increased IL-22-induced proliferation rates of PTC cells. Conversely, knockdown of TRIM30 resulted in inhibition of IL-22-induced proliferation of PTC cells (Fig. 3b). Similarly, TRIM30 overexpression increased IL-22-induced PTC cell migration and invasion, whereas TRIM30 knockdown decreased IL-22-induced PTC cell migration and invasion (Fig. 3c-f). We next investigated the effect of Sox17 on TRIM30-mediated proliferation of PTC cells. As shown in Fig. 3g, proliferation rates returned to control levels in Sox17-overexpressing cells. Conversely, knockdown of Sox17 enhanced TRIM30-induced PTC cell proliferation (Fig. 3h). Similar results were obtained in transwell migration and invasion experiments (Fig. 3i-l). This effect is not cell-type-specific because similar results were also obtained in KAT-5 cells (Additional file 1: Fig. S2). We next examined the effect of TRIM30 and Sox17 on the tumorigenicity in vivo. As shown in Fig. 3m-p, overexpression of TRIM30 promoted tumor growth in nude mice, whereas overexpression of Sox17 inhibited tumor growth. As expected, Sox17 overexpression counteracted the TRIM30-mediated tumor growth (Fig. 3m-p). These results indicate that the TRIM30/Sox17 axis mediates IL-22-induced proliferation in papillary thyroid cancer cells.

### IL-22 regulates β-catenin signaling through TRIM30/Sox17 axis

**Fig. 4:**
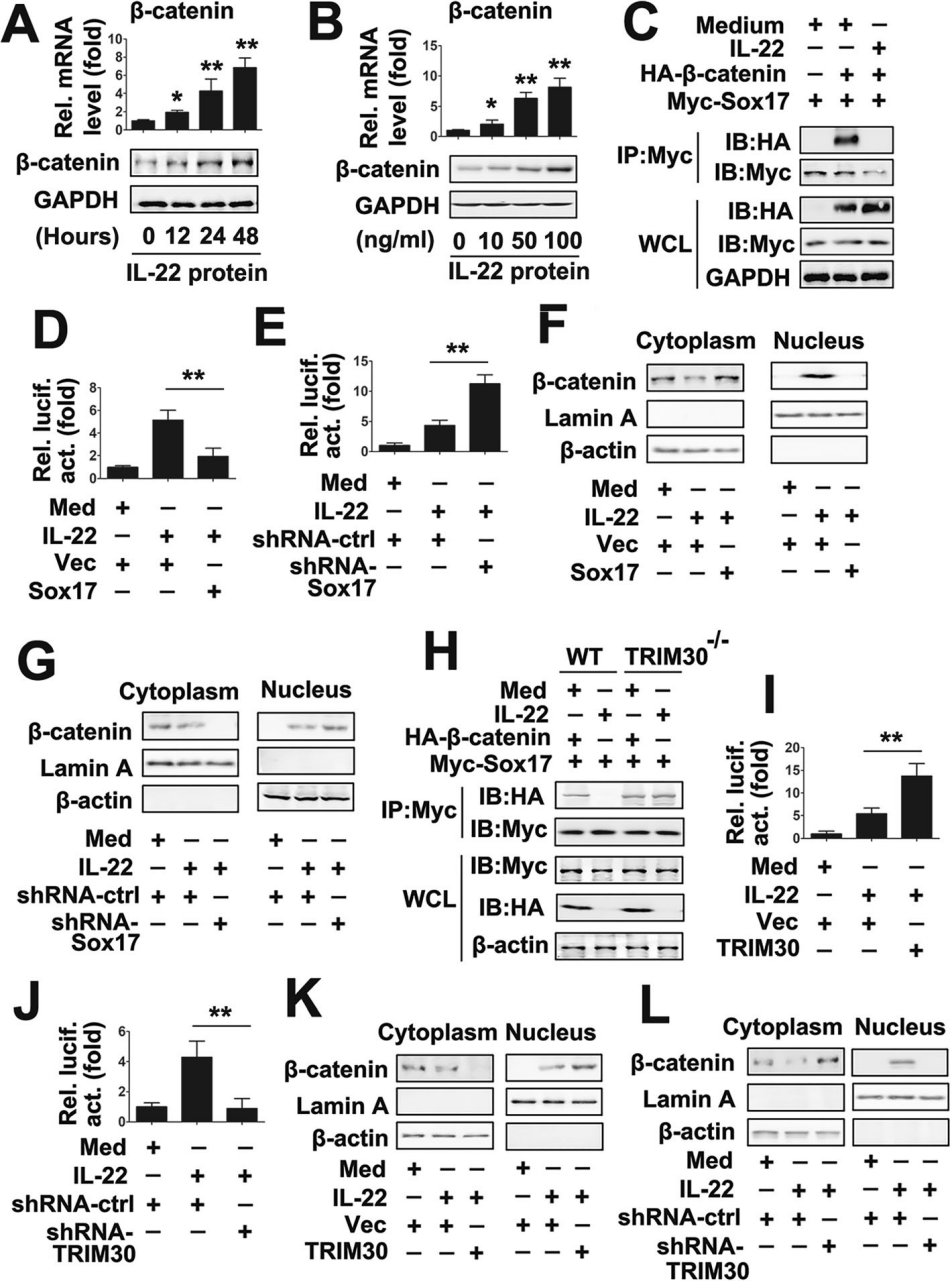
IL-22 regulate β-catenin signaling via TRIM30/Sox17 axis. (**a**) TPC-1 cells were treated with a concentration of 50 ng/ml rhIL-22 for the indicated time points. β-catenin levels were quantified by qRT-PCR (upper panel) and western blot (lower panel). (**b**) TPC-1 cells were treated with rhIL-22 for 24 h at indicated concentrations. β-catenin levels were quantified by qRT-PCR (upper panel) and western blot (lower panel). (**c**) TPC-1 cells transfected with the indicated plasmid for 24 h and treated with or without 50 ng/ml rhIL- 22 for 24 h. Co-IP and immunoblot analyses were performed with the indicated antibodies. (**d**) TPC-1 cells transfected with the indicated plasmid for 24 h and treated with or without 50 ng/ml rhIL- 22 for 24 h prior to luciferase assays. (**e**) Experiments were performed as in (**d**), except cells were transfected with shRNA-Sox17. (f) TPC-1 cells transfected with the indicated plasmid for 24 h and treated with or without 50 ng/ml rhIL- 22 for 24 h. Cytosolic and nuclear extracts were prepared and subjected to western blot analyses. Lamin A and β-actin were used as markers for nuclear and cytosolic fractions, respectively. (**g**) Experiments were performed as in (**f**), except cells were transfected with shRNA-Sox17. (**h**) Experiments were performed as in (**c**), except TRIM^−/−^ cells were used. (**i** and **j**) Experiments were performed as in (**d** and **e**), except pCMV-TRIM30 (i) or shRNA-TRIM30 (**j**) were used. (**k** and **l**) Experiments were performed as in (f and g), except pCMV-TRIM30 (**k**) or shRNA-TRIM30 (l) were used. All experiments were repeated at least three times. Bar graphs present means ± SD, *n* = 3 (**P < 0.01; *P < 0.05)

Because Sox17 interacts with β-catenin and promotes the degradation of β-catenin [24], we reasoned that IL-22 regulated β-catenin signaling. As shown in Fig. 4a and b, IL-22 also increased β-catenin mRNA and protein expression in a dose- and time-dependent manner in PTC cells. Further Western blot analyses revealed that IL-22 promoted the translocation of β-catenin, but not TRIM30 and Sox17, from the cytosol to the nucleus (Additional file 1: Fig. S3A). We also investigated the effect of IL-22 on the interaction of Sox17 and β-catenin. Co-IP experiments showed that IL-22 disrupted Sox17/β-catenin interactions (Fig. 4c). In addition, endogenous Co-IP experiments revealed that IL-22 disrupted Sox17/β-catenin interactions in cytoplasm (Additional file 1: Fig. S3B). We next investigated whether Sox17 involved in IL-22 regulated β-catenin signaling. Luciferase activity assays suggested that Sox17 overexpression decreased IL-22-induced β-catenin-luc activity, whereas TRIM30 knockdown increased IL-22-induced β-catenin-luc activity (Fig. 4d and e). Furthermore, we examined the effect of Sox17 on the translocation of β-catenin from the cytosol to the nucleus. Western blot analyses revealed that β-catenin protein levels were lowered in the cytosol and were elevated in the nucleus after IL-22 treatment (Fig. 4f and g). Overexpression of Sox17 prevented β-catenin translocation from the cytoplasm to the nucleus; conversely, knockdown of Sox17 induced β-catenin translocation from the cytoplasm to the nucleus (Fig. 4f and g).

Because TRIM30 is a Sox17-associated protein, we explored the possibility that TRIM30 mediated IL-22 regulated β-catenin signaling. As shown in Fig. 4h, IL-22 disrupted the Sox17/β-catenin interaction in TRIM30^+/+^ (WT) cells, but IL-22 did not regulate Sox17/β-catenin complex formation in TRIM30^−/−^ cells. Similarly, TRIM30 overexpression promoted IL-22-induced β-catenin-luc activity, whereas TRIM30 knockdown inhibited IL-22-induced β-catenin-luc activity (Fig. 4i and j). Consistently, overexpression of TRIM30 induced β-catenin translocation from the cytoplasm to nucleus; conversely, knockdown of TRIM30 prevented β-catenin translocation from the cytoplasm to the nucleus (Fig. 4k and l). Sox17 overexpression reduced IL-22-induced β-catenin inducible gene expression, whereas Sox17 knockdown induced IL-22-induced β-catenin inducible gene expression (Additional file 1: Fig. S4A and 4B). Conversely, TRIM30 overexpression promoted IL-22-induced β-catenin inducible gene expression, whereas TRIM30 knockdown inhibited IL-22-induced β-catenin inducible gene expression (Additional file 1: Fig. S4C and 4D). Taken together, these data suggest that IL-22-induces β-catenin signaling and that this is mediated through the TRIM30/Sox17 axis.

### TRIM30 regulates β-catenin signaling through Sox17

**Fig. 5:**
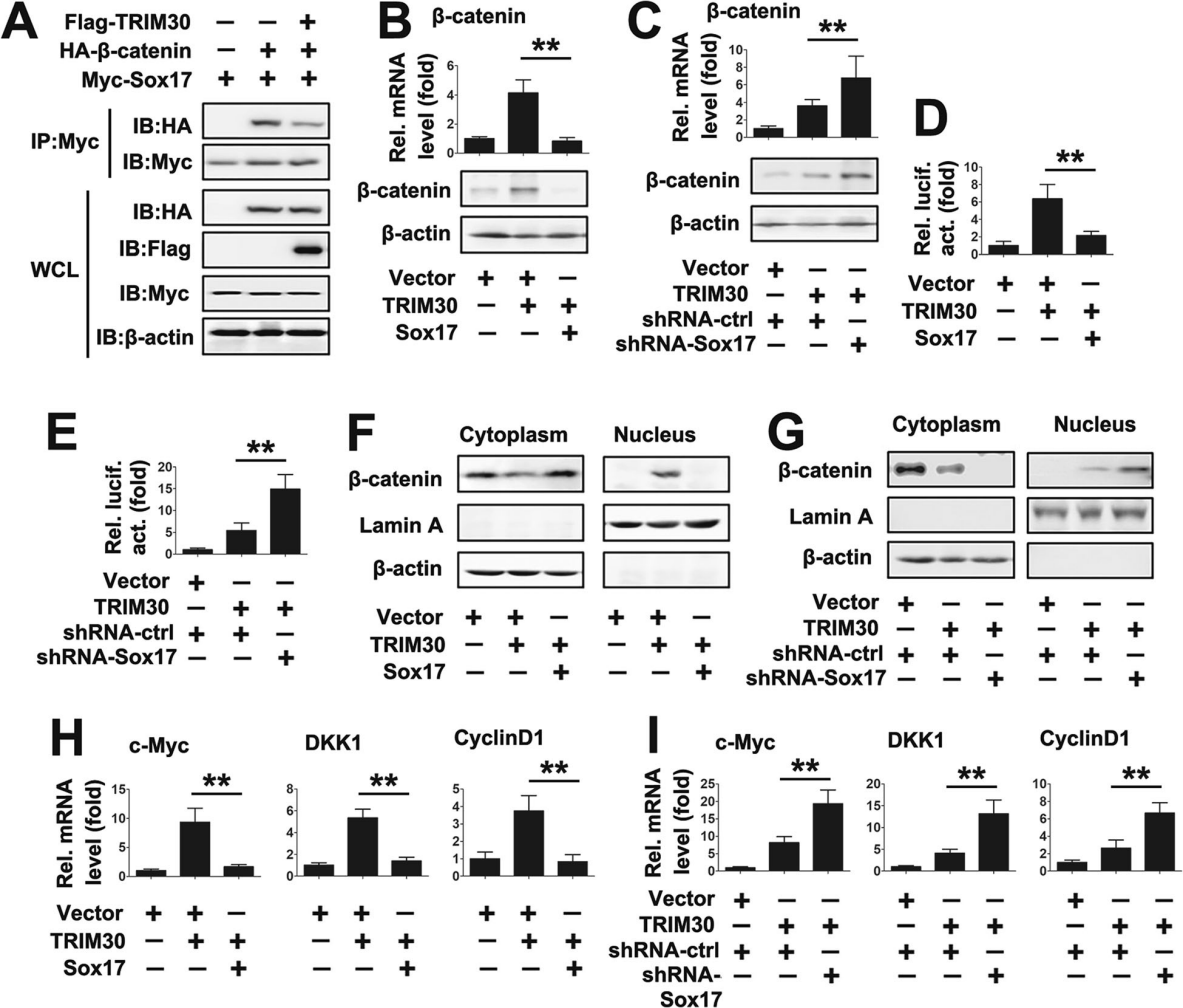
TRIM30 regulate β-catenin signaling via Sox17. (**a**) TPC-1 cells were transfected with Flag-TRIM30, Myc-Sox17 or HA-β-catenin for 48 h. Co-IP and immunoblot analysis were performed with the indicated antibodies. (**b**) TPC-1 cells were transfected with indicated plasmid for 48 h prior to qRT-PCR (upper panel) and western blot (lower panel). (**c**) Experiments were performed as in (**b**), except cells were transfected with shRNA-Sox17 (50 pmol). (**d**) TPC-1 cells were transfected with indicated plasmid for 48 h prior to luciferase assays. (**e**) Experiments were performed as in (**d**), except cells were transfected with shRNA-Sox17. (F) TPC-1 cells transfected with the indicated plasmid for 48 h. Cytosolic and nuclear extracts were prepared and subjected to western blot analyses. Lamin A and β-actin were used as markers for nuclear and cytosolic fractions, respectively. (**g**) Experiments were performed as in (**f**), except cells were transfected with shRNA-Sox17. (**h**) TPC-1 cells transfected with the indicated plasmid for 48 h prior to qRT-PCR assays. (**i**) Experiments were performed as in (**h**), except cells were transfected with shRNA-Sox17. All experiments were repeated at least three times. Bar graphs present means ± SD, *n* = 3 (**P < 0.01; *P < 0.05)

Because both TRIM30 and Sox17 are involved in IL-22 regulated β-catenin signaling, we reasoned that TRIM30 regulated β-catenin signaling through Sox17. As shown in Fig. 5a, TRIM30 overexpression disrupts Sox17/β-catenin interactions. We further examined the effects of TRIM30/Sox17 axis on β-catenin expression. qRT-PCR and western blot analyses showed that Sox17 abolished induction of TRIM30 on β-catenin expression (Fig. 5b). Conversely, the TRIM30 overexpression plasmid and the Sox17 shRNA synergistically induced β-catenin expression (Fig. 5c). Similarly, Sox17 overexpression decreased TRIM30-induced β-catenin-luc activity, whereas Sox17 knockdown increased TRIM30-induced β-catenin-luc activity (Fig. 5d and e). We examined the effect of Sox17 on TRIM30-regulated β-catenin nucleocytoplasmic transport. Western blot analyses showed that Sox17 overexpression decreased TRIM30-induced β-catenin nucleocytoplasmic transport, whereas Sox17 knockdown increased TRIM30-induced β-catenin nucleocytoplasmic transport (Fig. 5f and g). Consistent with this result, Sox17 overexpression reduced TRIM30-induced β-catenin inducible gene expression, whereas TRIM30 knockdown induced TRIM30-induced β-catenin inducible gene expression (Fig. 5h and i). Taken together, these results suggest that TRIM30 positively regulates β-catenin signaling by disrupting homotypic Sox17/β-catenin interactions.

### TRIM30 regulates Sox17 independent on miR-595

**Fig. 6:**
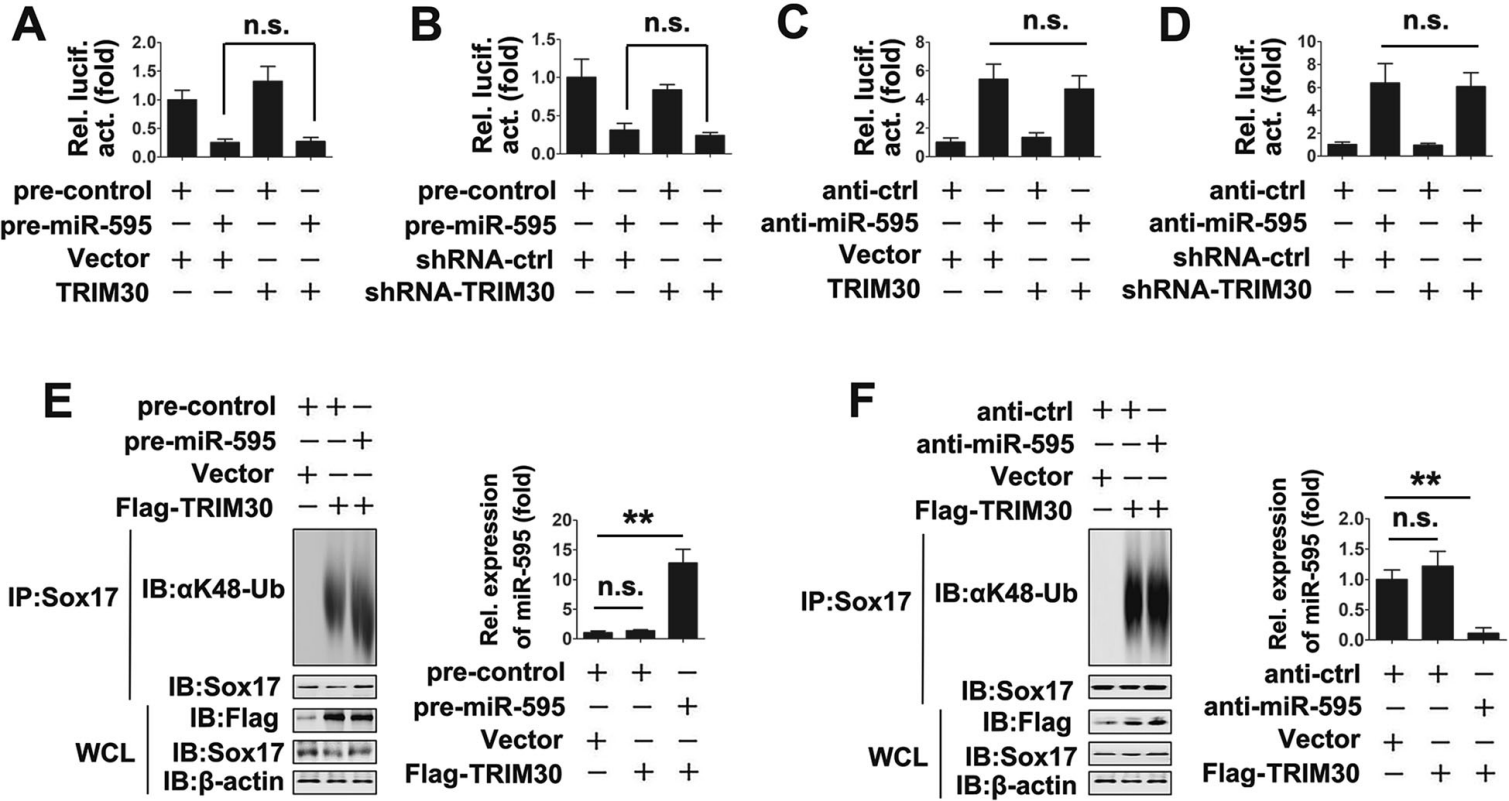
TRIM30 and miR-595 regulate Sox17 in a mutually independent manner. (**a**) TPC-1 cells were transfected with plasmids and miRNAs as indicated for 48 h prior to luciferase activity analyses. (**b**) Experiments were performed as in (**a**), except the shRNA-TRIM30 (50 pmol) were used. (**c** and **d**) Experiments were performed as in (**a** and **b**), except the indicated anti-miR-595 or anti-miR-control were used. (E) TPC-1 cells were transfected with the plasmids and miRNAs as indicated for 32 h. Co-IP and immunoblot analyses were performed with the indicated antibodies (left panel) and qRT-PCR was performed with the indicated primers. (**f**) Experiments were performed as in (**e**), except the indicated anti-miR-595 or anti-miR-control were used. All experiments were repeated at least three times. Bar graphs present means ± SD, *n* = 3 (**P < 0.01; *P < 0.05)

Because our previous study found that miR-595 regulated IL-22-induced PTC migration and invasion by binding to the 3′-UTR of Sox17, we determined the relationship between TRIM30 and miR-595. As shown in Fig. 6a and b, neither TRIM30 overexpression nor knockdown affected miR-595 inhibition of luciferase activity of the Sox17 3′-UTR. Consistent with this finding, TRIM30 overexpression or knockdown had no effect on the miR-595 inhibitor increased luciferase activity of the Sox17 3′-UTR (`. 6C and 6D). We also examined the effect of miR-595 on TRIM30-regulated Sox17 ubiquitination. As shown in Fig. 6e and f, both pre-miR-595 and anti-miR-595 did not affect TRIM30-regulated Sox17 ubiquitination. miR-595 expression detection was used to test transfection efficiency of pre-miR-595 or anti-miR-595 (Fig. 6e and f). Taken together, these results suggest that IL-22 induces PTC migration and invasion via TRIM30/Sox17 and miR-595/ Sox17, two independent signaling mechanisms.

### The expression of IL-22, TRIM30 and β-catenin in papillary thyroid tissues

**Fig. 7:**
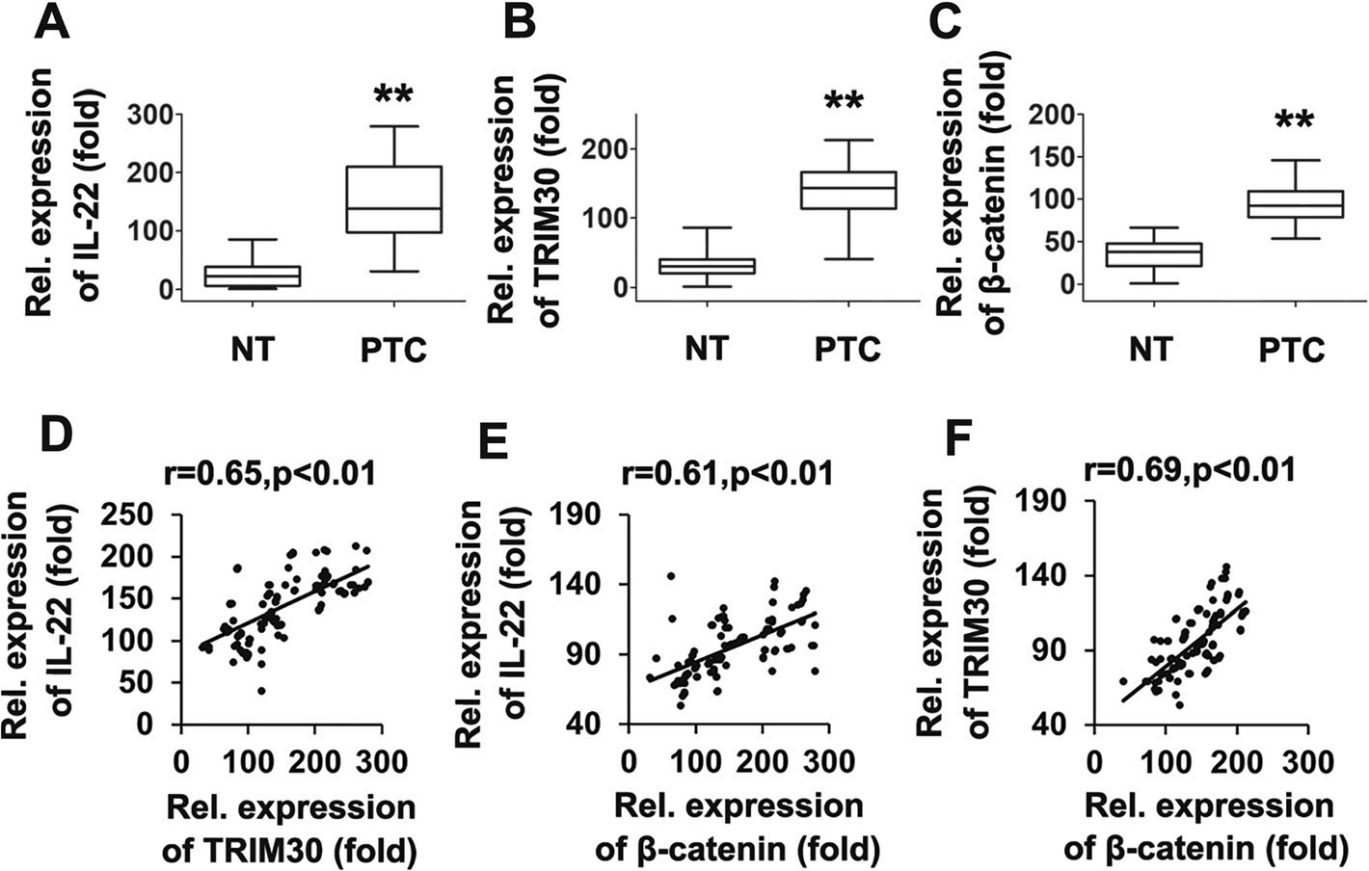
Expression IL-22, TRIM30, and β-catenin in papillary thyroid cancer tissues. (**a-c**) qRT-PCR experiments analyzing the expression of IL-22 (**a**), TRIM30 (**b**), and β-catenin (**c**) in 116 PTCs in comparison with the mean value obtained from 116 normal thyroid samples. (**d-f**) The relative IL-22 mRNA and TRIM30 levels (**d**), the relative IL-22 mRNA and β-catenin levels (**e**) and the relative TRIM30 mRNA and β-catenin levels (**f**) in the PTCs were subjected to Pearson’s correlation analysis. Box plots illustrate medians with 25th and 75th percentiles and error bars for 5th and 95th percentiles. For A-C, the lowest value was designated as 1. IL-22, TRIM30, and β-catenin data are expressed as fold-induction relative to the lowest value (**P < 0.01; *P < 0.05). The *p* values were calculated in SPSS 17.0 using Student’s t test

To further elucidate their roles in thyroid cancer, we determined expression levels of IL-22, TRIM30 and β-catenin in 116 pairs of clinical PTC and 116 adjacent nontumorous tissues (ANT). Using a qRT-PCR assay, we found that IL-22 was more highly expressed in PTC than in ANT (Fig. 7a), consistent with findings of our previous study. Consistent with the IL-22 data, TRIM30 and β-catenin expression levels were significantly higher than that of ANT (Fig. 7b and c). A statistically significant correlation was observed between expression levels of IL-22 and TRIM30, IL-22 and β-catenin, TRIM30 and β-catenin (Fig. 7d-f). These observations strongly suggest that alterations in IL-22, TRIM30 and β-catenin expression may be involved in thyroid cancer progression.

## Discussion

We described a novel mechanism for IL-22-regulated PTC cell migration and invasion. IL-22 promotes TRIM30 interaction with Sox17, thereby disrupting Sox17/β-catenin interactions. Further, studies showed that IL-22 induces PTC cell migration and invasion via the TRIM30/Sox17/β-catenin axis.

Sox17 is a member of the SRY-related high-mobility group (HMG)-box transcription factor superfamily [25]. SOX17 contains a conserved HMG box domain composed of three alpha helices and extended terminal tails adopting an L-shaped structure [26]. Apart from the independently folding HMG box, stretches outside the HMG box are poorly conserved and are composed of low-complexity regions with a high propensity to be intrinsically disordered, making them difficult to study [26]. Studies found that Sox17 participated in a variety of cell development processes and biological activities, including vascular development endoderm formation, oligodendrocyte development, and embryonic hematopoiesis [27, 28]. Specially, associated studies in animal models and tissue culture earned SOX17 the designation as ‘canonical WNT antagonist’ [27, 28]. In our previous study, we found that IL-22 induced miR-595 expression that in turn reduced Sox17 expression by directly targeting a specific binding site in the Sox17 3′-UTR, resulting in increased PTC cell migration and invasion [21]. In this study, we found that TRIM30 is a newly-discovered modulator of Sox17 in IL-22-regulated PTC cell migration and invasion. Interestingly, TRIM30/Sox17 and miR-595/Sox17 are two independent signaling pathways in IL-22 regulated PTC cell migration and invasion. Why does IL-22 need two regulators for Sox17? To our knowledge, this phenomenon appears to provide several forms of security for IL-22 to control molecules that play key roles in the IL-22-regulated signal pathway.

The tripartite motif (TRIM) protein family, most of which have E3 Ub ligase activity, includes over 70 highly-conserved proteins [29]. Members of the TRIM family usually contain a RING (R) domain, one or two B-box (B) domain(s) and a predicted coiled coil (CC) domain [30]. TRIM proteins have been reported to play important roles in antiviral immunity, inflammation and development. In recent years, the role of TRIM proteins in the development of cancer has attracted much attention. For example, TRIM47 overexpression promoted colorectal cancer cells proliferation and metastasis via ubiquitination and degradation of SMAD4 [31]. TRIM59 promoted breast cancer motility by targeting PDCD10. TRIM50 had tumor suppressor activity in hepatocellular carcinoma (HCC) cells by directly targeting SNAIL and reversing EMT [32]. TRIM44 promoted human esophageal cancer progression via the AKT/mTOR pathway [33]. Although the role of some members of TRIM family in cancer development have been clarified, the study of TRIM proteins in cancer motility (mode of migration and invasion) and metastasis remains largely uncharted territory. In this study, for the first time, we illustrated the mechanisms by which TRIM30 plays a role in PTC cell proliferation and metastasis. At the cellular level, we demonstrated that TRIM30 directly binds with Sox17 and induces K-48-linked poly-ubiquitination of Sox17 protein. Moreover, TRIM30/Sox17 axis also regulated tumor growth in nude mice. We further demonstrated that the function of β-catenin, a crucial downstream effector of canonical Wnt-β-catenin signaling during EMT, is positively regulated by TRIM30, and that TRIM30 might affect EMT by modulating Wnt-β-catenin signaling. Nevertheless, further studies are needed.

**Fig. 8:**
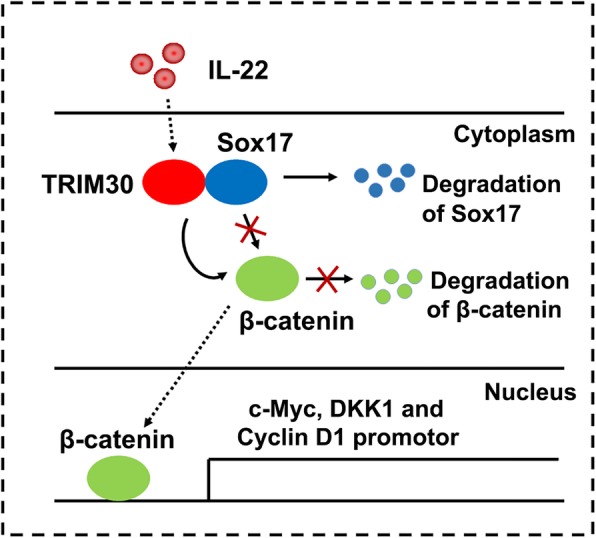
A hypothetical model for the role of TRIM30 in IL-22-regulated PTC cell migration and invasion. Under IL-22 treatment, TRIM30 associates with Sox17 for K48-linked polyubiquitination, thereby impairing the interaction between Sox17 and β-catenin. As a result, β-catenin translocates from the cytosol to the nucleus and activates downstream signaling, consequently contributing to PTC development

We propose a working model describing the role of TRIM30/Sox17/β-catenin axis in the migration and invasion of PTC cells (Fig. 8). In this model, treatment with IL-22 and TRIM30 interact with Sox17, leading to K48-linked ubiquitination and degradation of Sox17. As a result, Sox17 cannot ubiquitinate and degrade β-catenin or β-catenin translocation from the cytosol to the nucleus. Subsequently, β-catenin binds to promoters of the corresponding genes. In conclusion, Our data provide new knowledge regarding cancer motility and therapeutic strategies. Downregulating TRIM30- in Sox17-overexpressing cancers may create new avenues for treating papillary thyroid cancer.

## Supplementary information

**Additional file 1: Figure S1.** Determination of the efficiency of shRNAs and knockout (KO) cell lines. **Figure S2.** IL-22 promotes KAT-5 cells growth and motility via TRIM30/Sox17 axis. **Figure. S3.** Sox17 interact with TRIM30 and β-catenin in cytoplasm. **Figure. S4.** IL-22 regulate β-catenin inducible gene expression via TRIM30 and Sox17. **Table S1.** Correlation of IL-22, TRIM30 and β-catenin expression with clinicopathologic features in papillary thyroid cancers (PTC). **Table S2.** Antibodies used in this study. **Table S3.** Oligonucleotides Used in this study. **Table S4.** Other potential Sox17-interacting proteins identified by Co-IP and mass spectrometry.
