# Supplementary material for: TRIM30 modulates Interleukin-22-regulated papillary thyroid Cancer cell migration and invasion by targeting Sox17 for K48-linked Polyubiquitination
Source: Cell Commun Signal. 2019 Dec 10;17:162. doi: 10.1186/s12964-019-0484-6 (PMC6902597; doi:10.1186/s12964-019-0484-6)
Supplement: Supplementary file 1 — Additional file 1: Figure S1. Determination of the efficiency of shRNAs and knockout (KO) cell lines. Figure S2. IL-22 promotes KAT-5 cells growth and motility via TRIM30/Sox17 axis. Figure. S3. Sox17 interact with TRIM30 and β-catenin in cytoplasm. Figure. S4. IL-22 regulate β-catenin inducible gene expression via TRIM30 and Sox17. Table S1. Correlation of IL-22, TRIM30 and β-catenin expression with clinicopathologic features in papillary thyroid cancers (PTC). Table S2. Antibodies used in this study. Table S3. Oligonucleotides Used in this study. Table S4. Other potential Sox17-interacting proteins identified by Co-IP and mass spectrometry. [file 12964_2019_484_MOESM1_ESM.docx]

**TRIM30 Modulates** **Interleukin-22 Regulated** **Papillary Thyroid Cancer Cell Migration and Invasion by Targeting Sox17 for K48-linked Polyubiquitination**

Wei Li ^1^, Fen Li ^2^, Weiwei Lei^1^, Zezhang Tao ^1,2^ *


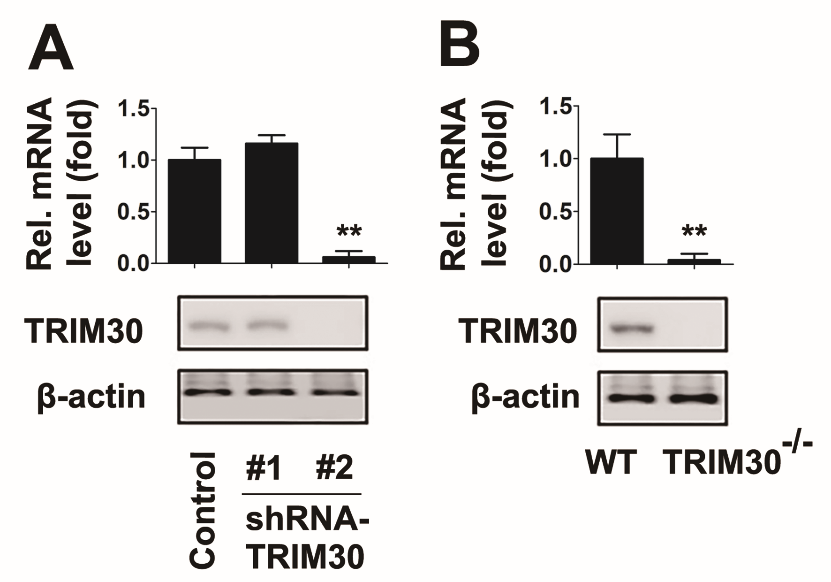


**Supporting Fig. 1. Determination of the efficiency of shRNAs and knockout (KO) cell lines.** (A) TPC-1 cells were transfected with shRNA control or TRIM30-specific shRNAs for 48 h prior to qRT-PCR (upper panel) and western blot (lower panel). (B) TPC-1 cells were used to construct TRIM30-KO (TRIM30^-/-^) cell lines using the CRISPR-Cas9 system. TPC-1 wild-type cells (TRIM30^+/+^) and monoclonal KO cells were examined using qRT-PCR (upper panel) and western blot (lower panel). All experiments were repeated at least three times with consistent results. Bar graphs represent the means ± SD, n = 3 (**P < 0.01; *P < 0.05).


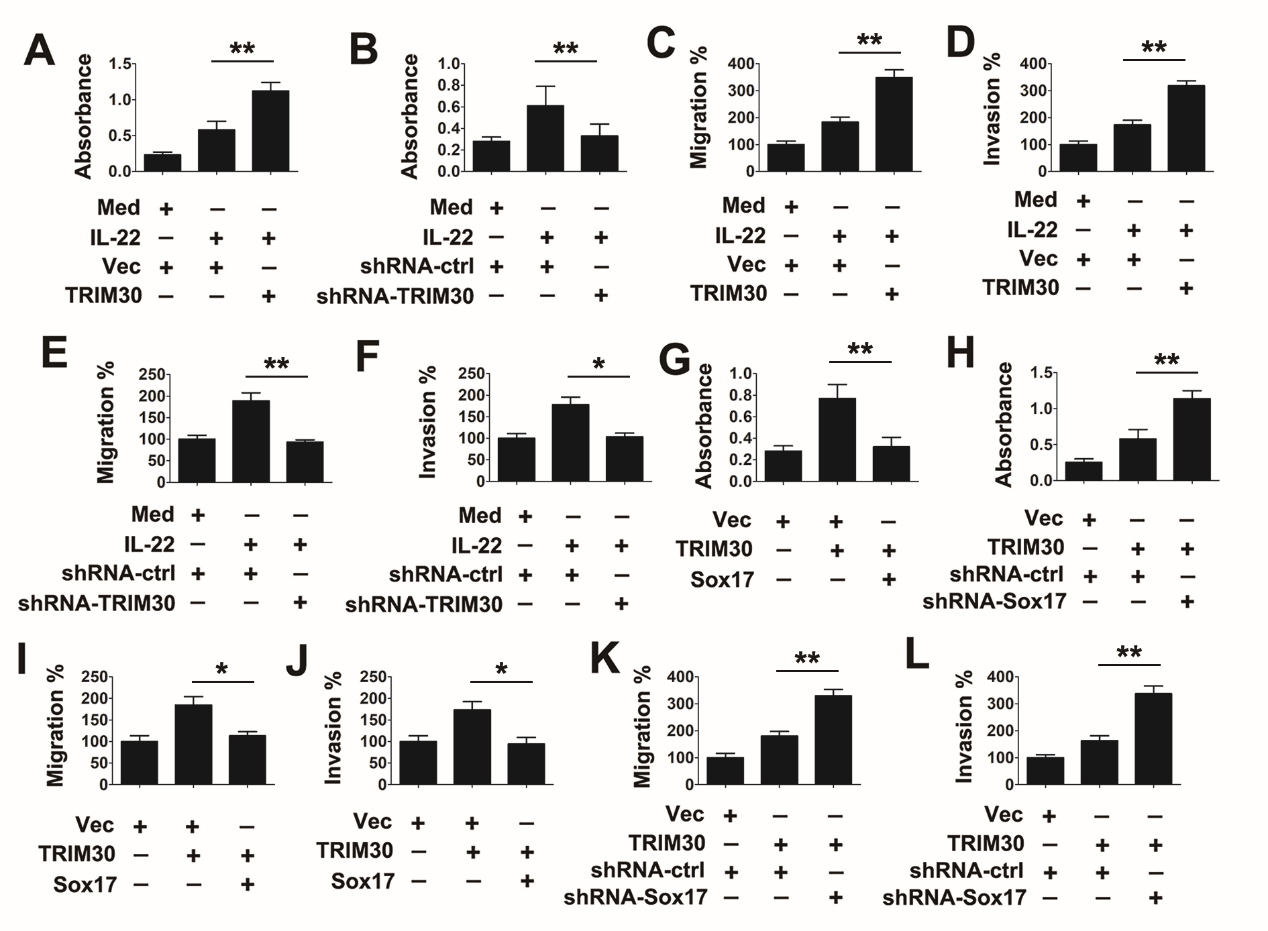


**Supporting Fig. 2. IL-22 promotes KAT-5 cells growth and motility via TRIM30/Sox17 axis.** Experiments were performed as in Fig 3, except KAT-5 cells were used. Bar graphs present means ± SD, n = 3 (**P < 0.01; *P < 0.05).


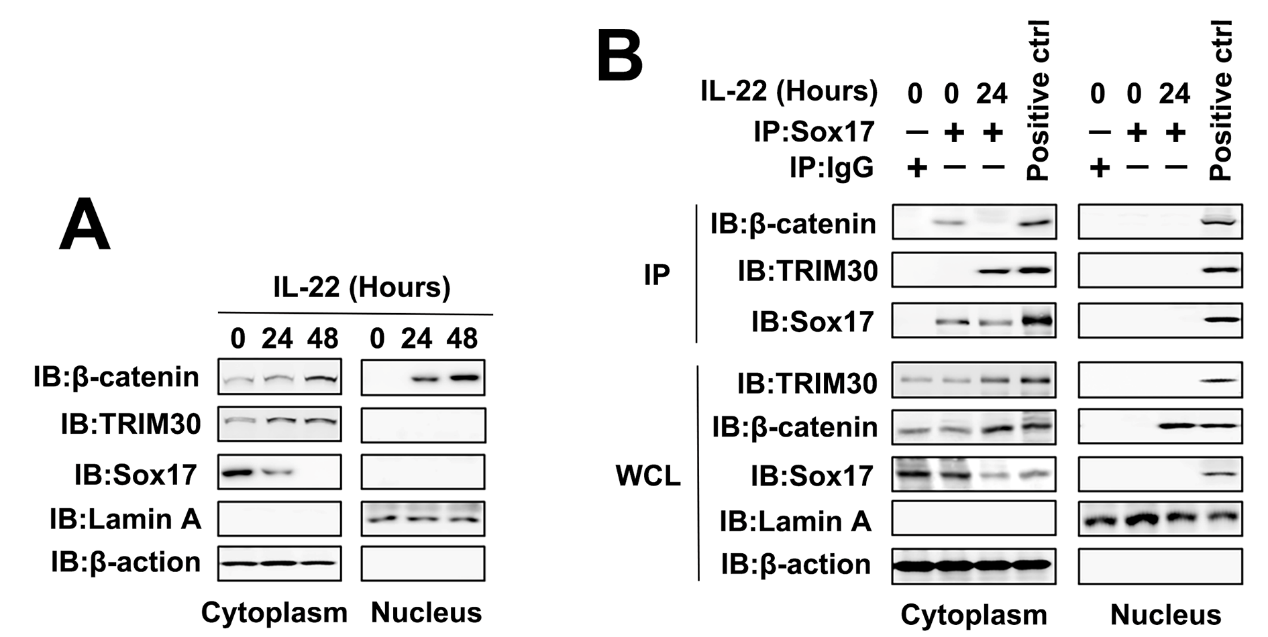


**Supporting Fig. 3. Sox17 interact with TRIM30 and β-catenin in cytoplasm.** (A) TPC-1 cells treated with or without 50 ng/ml rhIL- 22 for indicated times. Cytosolic and nuclear extracts were prepared and subjected to western blot analyses. Lamin A and β-actin were used as markers for nuclear and cytosolic fractions, respectively. (B) TPC-1 cells treated with or without 50 ng/ml rhIL- 22 for indicated times. Cytosolic and nuclear extracts were prepared and Co-IP and immunoblot analyses were performed with the indicated antibodies. Whole cell lysate was used as positive control. All experiments were repeated at least three times.


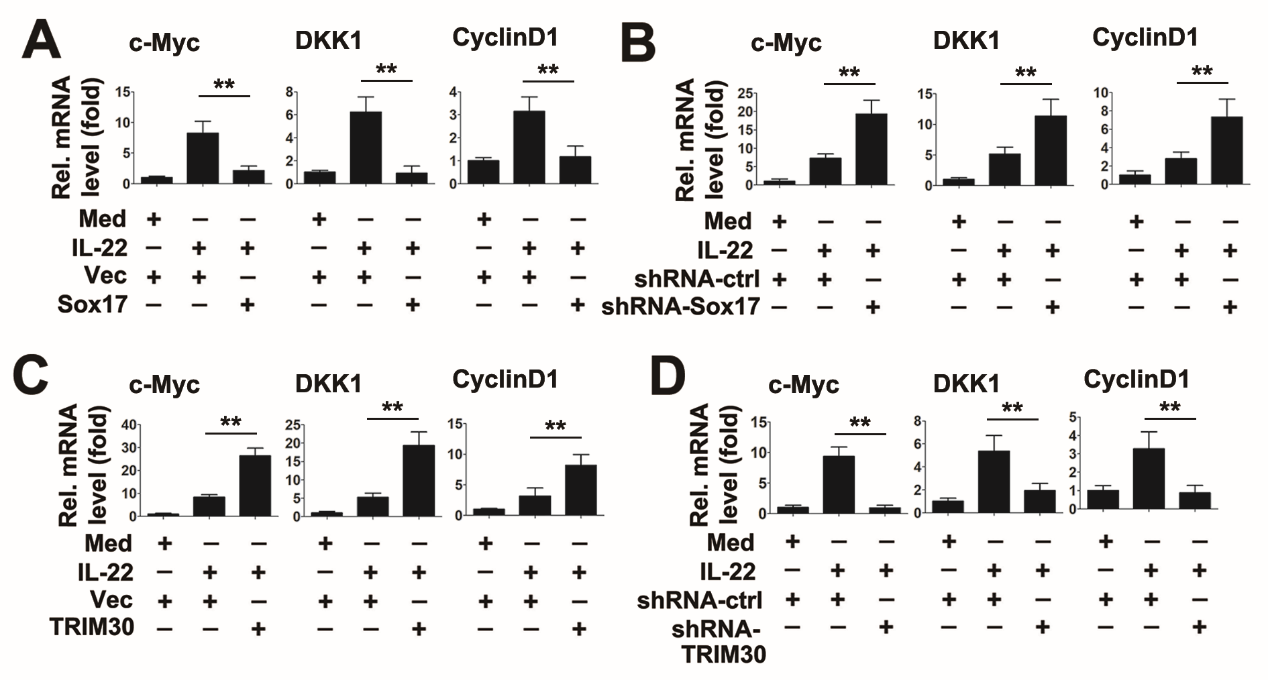


**Supporting Fig. 4. IL-22 regulate β-catenin inducible gene expression via TRIM30 and Sox17.** (A) TPC-1 cells transfected with the indicated plasmid for 24 h and treated with or without 50 ng/ml rhIL- 22 for 24 h prior to qRT-PCR assay. (B) Experiments were performed as in (D), except cells were transfected with shRNA-Sox17. (C and D) Experiments were performed as in (A) and (B), except cells were transfected with TRIM30 plasmid (C) or shRNA-Sox17 (D). Bar graphs present means ± SD, n = 3 (**P < 0.01; *P < 0.05).

**Supplemental Table 1: Correlation of IL-22, TRIM30 and β-catenin expression with** **clinicopathologic features in papillary thyroid cancers (PTC).**

| **Clinicopathologic parameters** | **Case no.** | **IL-22 expression (folds)** | **P value** | **TRIM30 expression(folds)** | **P value** | **β-catenin expression (folds)** | **P value** |
| --- | --- | --- | --- | --- | --- | --- | --- |
| **Age** |  |  |  |  |  |  |  |
| ≤50 | 52 | 155.36±11.74 | ns | 143.76±14.95 | ns | 91.27±11.29 | ns |
| ＞50 | 64 | 147.88±15.96 |  | 137.63±13.71 |  | 97.63±12.41 |  |
| **Tissue type** |  |  |  |  |  |  |  |
| Normal tissue | 116 | 24.04±3.36 | <0.01 | 31.06±6.74 | <0.01 | 33.69±4.11 | <0.01 |
| Carcinoma | 116 | 151.6±19.33 |  | 140.55±19.35 |  | 94.45±11.07 |  |
| **Sex** |  |  |  |  |  |  |  |
| Male | 64 | 150.84±16.73 | ns | 137.84±11.25 | ns | 96.33±9.34 | ns |
| Female | 52 | 152.74±18.69 |  | 143.96±14.97 |  | 92.18±7.18 |  |
| **Tumor size** |  |  |  |  |  |  |  |
| ≤5cm | 55 | 91.24±10.25 | <0.01 | 88.26±9.21 | <0.01 | 64.17±8.77 | <0.01 |
| ＞5cm | 61 | 211.85±26.93 |  | 192.37±24.97 |  | 124.93±13.04 |  |
| **TNM stage** |  |  |  |  |  |  |  |
| Ⅰ | 23 | 43.96±6.37 | <0.05 | 36.25±4.17 | <0.05 | 23.57±3.69 | <0.05 |
| Ⅱ | 31 | 84.15±9.17 |  | 79.11±9.36 |  | 47.48±3.17 |  |
| Ⅲ | 33 | 172.15±15.37 |  | 141.25±10.24 |  | 92.34±8.15 |  |
| Ⅳ | 29 | 305.95±27.84 |  | 306.17±29.34 |  | 211.74±17.48 |  |
| **Lymph nodemetastasis** |  |  |  |  |  |  |  |
| Negative | 56 | 75.69±8.63 | <0.01 | 68.27±4.17 | <0.01 | 39.26±5.27 | <0.01 |
| Positive | 60 | 227.97±24.74 |  | 212.58±16.38 |  | 149.14±18.95 |  |
| **Distant metastasis** |  |  |  |  |  |  |  |
| Negative | 57 | 91.87±10.74 | <0.01 | 75.87±8.37 | <0.01 | 36.27±5.27 | <0.01 |
| Positive | 59 | 211.96±26.37 |  | 205.37±22.74 |  | 152.33±16.78 |  |

**Supplemental Table 2: Antibodies used in** **this study**

| Antibodies | Source | Identifier |
| --- | --- | --- |
| HA | Sigma | Cat#H6908 (1:2000) |
| Flag | Sigma | Cat#M2 (1:2000) |
| Myc | Abcam | Cat#ab32 (1:2000) |
| β-actin | Abcam | Cat#ab179467 (1:5000) |
| TRIM30 | Abcam | Cat# ab76953 (1:1000) |
| Sox17 | Abcam | Cat# ab224637 (1:500) |
| Lamin A | Cell Signaling Technology | Cat# 86846 (1:1000) |
| β-catenin | Cell Signaling Technology | Cat# 8480 (1:1000) |
| K48-linkage polyubiquitin | Cell Signaling Technology | Cat#8081 (1:1000) |

**Supplemental Table 3: Oligonucleotides Used in this study**

| **Target gene** | **5’primer (5’ to 3’)** | **3’primer (5’ to 3’)** |
| --- | --- | --- |
| **Primers** | | |
| **GAPDH** | GGAAGGTGAAGGTCGGAGTCAACGG | CTCGCTCCTGGAAGATGGTGATGGG |
| **IL-22** | CCCCAGTCACCAGTTGCTCG | AGGGCTGCTGGAAGTTGGAC |
| **Sox17** | GGATACGCCAGTGACGACC | CGACTTGCCCAGCATCTT-3 |
| **β-catenin** | TCAGAGCAGGTACAAGGGTA | AATGTCTCAGGGAACATAGC |
| **c-Myc** | AACTTACAACACCCGAGCAA | CGAGTCGTAGTCGAGGTCATAG |
| **DKK1** | AGCGTTGTTACTGTGGAGAA | CATTTGGATAGCTGGTTTAG |
| **CyclinD1** | TTCCTGTCCTACTACCGCCTCA | CCCTCAGATGTCCACGTCCC |
| **shRNAs** | | |
| **Genes** | Target sequence (5’ to 3’) | |
| **TRIM30#1** | CAGCUCUCAUUGAAGAGGUTT | |
| **TRIM30#2** | GGAGAAUGAGGAGCUGCAGTT | |
| **Sox17** | GCATCGCTGGGTTTAAGATAAAGGA | |
| **control** | CAACTATCCTGACGTGTGACAGGTC | |
| **KO** **target sequence** | | |
| **Trim30** | TTCATATGACTCTACATGCAAGG | |

**Supplemental Table 4: Other potential Sox17-interacting proteins identified by Co-IP and mass spectrometry.**

| Identified proteins | Peptides |
| --- | --- |
| RFC1 (Replication factor C subunit 1) | K.IGEVSSPK.A |
|  | K.AALLSGPPGVGK.T |
|  | R.GGIQELIGLIK.H |
|  | R.TVNMDYLSLLR.D |
|  | K.AIVAESLNNTSIK.G |
|  | K.IIDEDGLLNLIR.T |
|  | K.LTPTSVLDYFGTGSVQR.S |
| HRNR (Hornerin) | R.GPYESGSGHSSGLGHR.E |
|  | R.GSGSGQSPSSGQHGTGFGR.S |
|  | R.QSLGHGQHGSGSGQSPSPSR.G |
|  | R.HGSGSGQSSSYSPYGSGSGWSSSR.G |
|  | R.HGSGSGHSSSYGQHGSGSGWSSSSGR.H |
| RAE1 (mRNA export factor) | R.NAAEELKPR.N |
|  | R.GLIVYQLENQPSEFR.R |
| ILF3 (Interleukin enhancer-binding factor 3) | R.IFVNDDR.H |
|  | K.FNYSGSGGR.S |
|  | K.AYAALAALEK.L |
|  | K.VLGMDPLPSK.M |
|  | K.EATDAIGHLDR.Q |
|  | R.LNQLKPGLQYK.L |
|  | K.LFPDTPLALDANK.K |
|  | K.VLQDMGLPTGAEGR.D |
|  | K.LFPDTPLALDANKK.K |
|  | K.VLAGETLSVNDPPDVLDR.Q |
| Nup93 (Nuclear pore complex protein Nup93) | K.LYDLAK.N |
|  | K.ASVLLGSR.G |
|  | R.AFDIIER.L |
|  | R.NLQEIQQAGER.L |
|  | R.SSLDNIEMAYAR.Q |
|  | R.TLITFAGMIPYR.T |
|  | K.QMTDVLLTPATDALK.N |
|  | K.LLSPVVPQISAPQSNK.E |
| CCAR2 (Cell cycle and apoptosis regulator protein 2) | R.FSATEVTNK.T |
|  | K.VQTLSNQPLLK.S |
|  | K.VLLLSSPGLEELYR.C |
| DNMT3A (DNA (cytosine-5)-methyltransferase 3A) | K.EIIDER.T |
|  | K.ETNIESMK.M |
|  | K.IMYVGDVR.S |
|  | R.LFFEFYR.L |
|  | R.GFGIGELVWGK.L |
|  | K.AIYEVLQVASSR.A |
|  | R.FLESNPVMIDAK.E |
|  | R.KAIYEVLQVASSR.A |
|  | R.LTFQAGDPYYISK.R |
|  | K.GGAPAEGEGAAETLPEASR.A |
| MCM2 (DNA replication licensing factor MCM2) | K.AGIVTSLQAR.C |
|  | R.GLALALFGGEPK.N |
|  | R.ISHLPLVEELR.S |
|  | R.DNNELLLFILK.Q |
|  | R.DTVDPVQDEMLAR.F |
| Jun (Transcription factor Jun) | K.LASPELER.L |
|  | K.NSDLLTSPDVGLLK.L |
|  | K.NVTDEQEGFAEGFVR.A |
| DSP (Desmoplakin) | K.IEVLEEELR.L |
|  | R.AELIVQPELK.Y |
|  | R.YEVTSGGGGTSR.M |
|  | R.ETQSQLETER.S |
|  | R.LNDSILQATEQR.R |
| TIF1B (Transcription intermediary factor 1-β) | R.FFETR.M |
|  | R.VLVNDAQK.V |
|  | R.MNEAFGDTK.F |
|  | K.IVAERPGTNSTGPAPMAPPR.A |
|  | M.AASAAAASAAAASAASGSPGPGEGSAGGEKR.S |
|  | K.LTEDKADVQSIIGLQR.F |
|  | M.AASAAAASAAAASAASGSPGPGEGSAGGEK.R |
|  | M.AASAAAASAAAASAASGSPGPGEGSAGGEKR.S |
| ATP1A1 (Sodium/potassium-transporting ATPase subunit α-1) | R.AAEILAR.D |
|  | R.AAVPDAVGK.C |
|  | R.AVAGDASESALLK.C |
|  | R.LNIPVSQVNPR.D |
|  | K.VDNSSLTGESEPQTR.S |
|  | K.GVGIISEGNETVEDIAAR.L |
| FLG2 (Filaggrin-2) | R.SVVTVIDVFYK.Y |
|  | R.FSNSSSSNEFSK.C |
|  | R.HQEEESETEEDEEDTPGHK.S |
|  | R.SGQSSYGQHSSGSSQSSGYGQHGSR.Q |
